# Flowable Urethane Dimethacrylate-Based Filler for Root Canal Obturation in Primary Molars: A Pilot SEM and microCT Assessment

**DOI:** 10.3390/children8020060

**Published:** 2021-01-20

**Authors:** Saulius Drukteinis, Audra Drukteiniene, Lesbia Drukteinis, Luc C. Martens, Sivaprakash Rajasekharan

**Affiliations:** 1 Institute of Dentistry Faculty of Medicine Vilnius University, Zalgirio 115, LT-08217 Vilnius, Lithuania; 2 Children’s Hospital, Affiliate of Vilnius University Hospital Santaros Klinikos, Santariskiu 7, LT-08406 Vilnius, Lithuania; audra.drukteiniene@santa.lt; 3 Department of Growth, Development and Structure, Section of Pediatric Dentistry, Southern Illinois University, School of Dental Medicine, Alton, IL 62002, USA; ledrukt@siue.edu; 4 Department of Paediatric Dentistry, Ghent University School of Oral Health Sciences, B-9000 Ghent, Belgium; luc.martens@ugent.be (L.C.M.); sivaprakash.rajasekharan@ugent.be (S.R.)

**Keywords:** micro-computed tomography, primary molar, primary teeth, root canal filling, root canal treatment, scanning electron microscopy

## Abstract

Pulpectomy in deciduous teeth involves endodontic access opening, root canal debridement and obturation with an appropriate filling material. EndoREZ (ER) is the urethane dimethacrylate-based filler, which can be used for root canal obturation in permanent and primary teeth. This observation aimed to evaluate the behavior of the ER as a filler in root canals of two primary molars after the physiological resorption process using the scanning electron microscopy (SEM) and micro-computed tomography (µCT) in second lower molars after their natural exfoliation. The SEM analysis revealed a non-uniform, porous and lacunary structure of ER, visually similar to the resorbed surface of the dentine. The µCT observations demonstrated the differences in the resorption level of the root and material surfaces. The preliminary observations suggest that ER is resorbed faster than root tissues and can therefore be a suitable material for the root canal filling in primary teeth. However, more investigations are needed to support these preliminary findings.

## 1. Introduction

The recent micro-computed tomographic (µCT) studies discovered the complexity and anatomical diversity of the roots and root canals in primary teeth [1], making the endodontic treatment very challenging for the clinicians. Nonvital pulp therapy in primary teeth has substantial limitations and can be considered just if the pulp tissue is inflamed irreversibly, is necrotic and infected [2]. Additionally, the roots of the teeth should have minimal or no signs of resorption [3]. Vital pulp therapy in primary teeth relies on pulpotomy procedures, including the recent use of hydraulic calcium silicate-based materials [4]. However, when pulpotomy is not indicated, the pulpectomy can be the best option to preserve the affected tooth in the primary dentition. Alternatively, extractions may require additional investments into subsequent use of the space maintainers for the erupting permanent teeth. These fixed and removable space maintainers can also negatively affect oral hygiene, increase the plaque accumulation, which can lead to demineralization, caries or periodontal problems [5].

If indicated, pulpectomy involves endodontic access opening, root canal debridement via instrumentation irrigation, disinfection and obturation with an appropriate filling material [6,7]. The ideal root canal filling material for primary teeth should be resorbable and have no effect on the physiological root resorption [7]. Moreover, it should be antibacterial and biocompatible while not having any harmful effect on the permanent tooth germ if extruded periapically [8]. The whole range of paste-type materials was suggested for root canal filling in primary teeth, such as zinc oxide-eugenol (ZOE), calcium hydroxide with or without iodoform, iodoform based and others. [7].

Meanwhile, the new urethane dimethacrylate-based (UDMA) material EndoREZ (ER) (Ultradent Products Inc., South Jordan, UT, USA) was suggested to be used for the obturation of the root canals in primary teeth, due to the material’s hydrophilicity, acceptable biocompatibility, low cytotoxicity and enhanced resorbability in contact with vital tissues [9,10,11,12,13]. The material belongs to the second generation of the methacrylate-based materials, is a dual cure and was launched as a sealer/filler for root canal obturation with different obturation techniques [14,15]. The material is launched in a dual-syringe, containing base and catalytic pastes, which are mixed using mixing tips and delivered to the root canal using injection technique [14]. The fast and simplified root canal obturation via injection can be particularly beneficial in pediatric dentistry due to ease of delivery.

Despite the numerous published studies, analyzing important physicochemical, biological properties and clinical performance of the ER in permanent teeth [9,12,15], there is no data available regarding the effectiveness and performance of the material as a root canal filler in the primary teeth undergoing physiological root resorption at the microscopic and two- or three-dimensional (2D, 3D) level. Therefore, to provide some evidence-based and objective information on the behavior of the ER in root canals of primary molars after the physiological resorption, the scanning electron microscopic (SEM) and µCT observation of the naturally exfoliated second lower molars was conducted.

## 2. Materials and Methods

**Figure 1:**
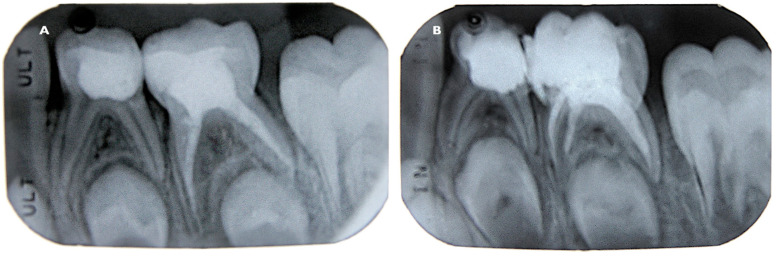
Postoperative radiographs of two mandibular second primary molars after endodontic treatment and coronal restoration (**A**,**B**).

Two mandibular second primary molars, naturally exfoliated due to the physiological root resorption, were voluntarily provided by two patients and their parents for evaluation. The patient consent forms were signed before investigation and publication of the data. According to the dental records, the irreversible pulpitis was diagnosed in both cases, and the single visit pulpectomy under rubber-dam isolation was performed by the specialist-pediatric dentist on mandibular primary second molars with each patient at age four. In each case conventional pulpectomy using 1% NaOCl as an irrigant was performed, followed by filling the debrided and disinfected root canals with urethane dimethacrylate-based sealer/filler ER using the injection technique. The material was delivered using Skinni syringe (Ultradent Products Inc.) and 29-G NaviTip needles (Ultradent Products Inc.). The root canal orifices and pulp chamber floor were covered with Fuji IX (GC Corporation, Tokyo, Japan) glass-ionomer cement, while the remaining endodontic access was filled with compomer Dyract (Dentsply DeTrey GmbH, Konstanz, Germany) providing a restoration that seals the tooth from microleakage (Figure 1A,B).

There were no reported patient complaints, clinical symptoms or pathological radiographic changes in periodontal tissues during the five years observation period. Teeth were naturally exfoliated due to physiologic root resorption. The debris was removed submerging teeth into 1% of NaOCl for 5 min and distilled water. Cleaned teeth were subjected to SEM and microCT observation.

Characterization of the resorbed roots surfaces was performed by SEM (T3030; Hitachi Ltd., Tokyo, Japan) under vacuum and at 15 kV. The magnifications of ×40, ×80, ×400, ×500, ×800 and ×1000 were used to observe different zones of the roots, roots-material interface and material surfaces, to detect specific profile features.

The scanning of primary molars was performed using a high-resolution µCT scanner SkyScan 1173 (Bruker-microCT, Kontich, Belgium) preset with the following main parameters: 110 kV, 50 μA and an image pixel size of 22.8 μm. Moreover, 1-mm aluminium filter, 180° rotation and a rotation step of 0.45 were adjusted. The scanned images were reconstructed with ring artifact correction factor of 7, beam hardening correction of 26% using NRecon v.1.6.9 (Bruker-microCT) software, while the scanning data were analyzed using CTAn v.1.14.4 software (Bruker-microCT).

## 3. Results

**Figure 2:**
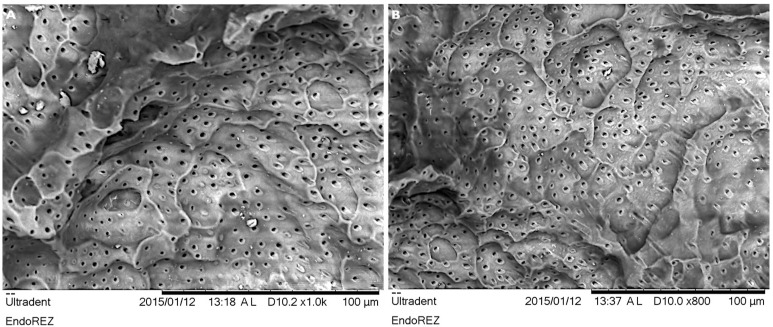
The typical surface profile of the resorbed root of primary teeth with lacune-type dentin defects at ×1000 (**A**) and ×800 (**B**) magnification.

**Figure 3:**
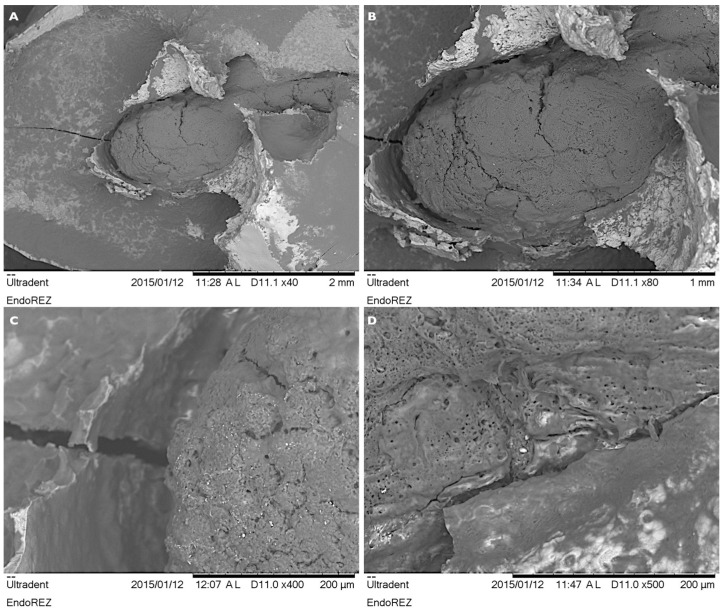
The appearance of ER filler localized in the center of the resorbed root at ×40 (**A**) and ×80 (**B**) magnification. The porous, resorbed surface profile of the ER material at magnifications of ×400 (**C**) and ×500 (**D**).

The results of SEM investigations are displayed and visualized in Figure 2 and Figure 3. The observed dentine surfaces demonstrated multiple lacune-like resorptive defects, which are representative of the typical external root resorptions (Figure 2A,B). The numerous wide and less regular dentinal tubules were clearly visible, indicating and reflecting morphological features of dentine of the primary teeth.

At the lower magnifications of ×40 and ×80, the ER filler was visible in the center of the field of view and repeated the outline contours of the canal of the resorbed root (Figure 3A,B). Visually the material appeared deeper in the canal, demonstrating the different resorption levels of the ER and root dentine surfaces. The wide gaps between the filler mass and the root canal wall were visible, while the edges of the filler at the material-gap-dentin interface were rounded. Multiple crack lines and perpendicular defects were identified on the surface of the material. At the higher magnifications (Figure 3C,D), the ER material exhibited non-uniform, porous and lacunary structure, visually similar to the resorbed surface of the dentine.

**Figure 4:**
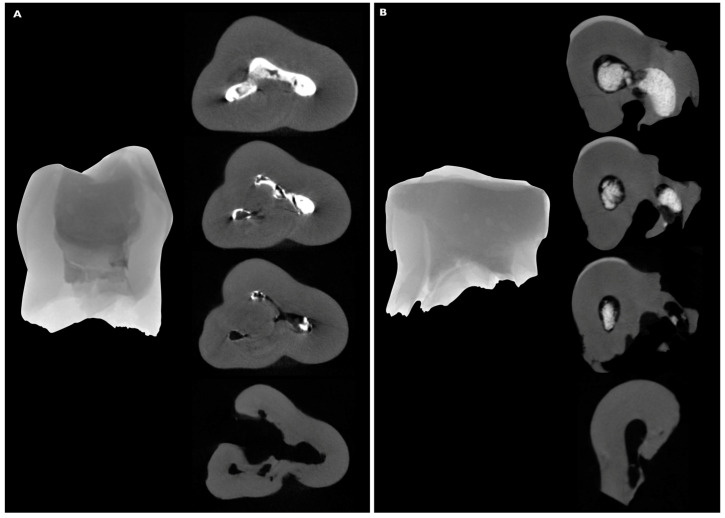
The representative images of the two scanned molars (**A**,**B** left) and their cross-sectional images (**A**,**B** right) at the different levels of the resorbed roots, demonstrating the presence/absence of the ER filler inside the root canal space.

The scanned primary molars and their cross-sectional images after reconstruction are shown in Figure 4.

The µCT observations revealed the possible differences in the response of the root hard tissues and ER filler during the physiological resorption process, and these differences were evident in both scanned teeth. The differences were related to the level of the resorption: the surface of the resorbed ER material was below the root surface, while the average distance was 3.12 mm (molar A) and 2.17 mm (molar B), respectively. Despite the oblique resorption nature and different resorption intensity for M and D roots (Figure 4B), the same predominance of more extensive ER material resorption over root structures was observed.

## 4. Discussion

The success rates of pulpectomy in primary teeth is relatively high and varies from 60% up to 98% [8]. However, it should be clearly highlighted, that it can be achieved just when strict case selection procedures are performed before treatment, as the overall indications for pulpectomy procedures in primary dentition are quite limited [16]. The successful pulpectomy in primary teeth is considered, when there are no clinical signs of periodontal disease, the tooth is painless, mobility is normal, surrounding soft tissues are without evidence of inflammation [8]. Radiographically, the preexisting radiolucency/lesion is not increasing or is decreasing, no new lesions are detected either periapically or in furcation area are not detected, and the physiological root resorption is visible [6,17].

It has been demonstrated, that pulpectomy outcomes differs significantly due to the material used for root canal filling: the lowest rates were detected when calcium hydroxide paste was used, while better rates were associated with iodoform, zinc oxide-iodoform or zinc oxide-eugenol formulations [17,18]. The overfilling of the root canals and capability of the materials to be relatively quickly resorbed after extrusion have a direct impact on treatment outcome [8]. The extruded material can induce foreign body-type inflammatory reaction or, being not well resorbable, can alter the pathway for permanent teeth to erupt or even to arrest the eruption [7]. Moreover, numerous cases of ectopic eruption of the deciduous teeth were demonstrated after ZOE pulpectomy [19]. Additionally, it has been demonstrated, that many filling materials, used in pediatric endodontics did not resorb as expected and remnants of the materials are detectable on radiographs even several months after exfoliation of endodontically treated primary tooth [7,8].

The potential of ER to be resorbed has been widely evaluated in animal studies and long-term clinical trials in permanent teeth [9,10,20,21]. It has also been shown that even if extruded periapically the material is well tolerated by connective and bone tissues [10,12]. The most interesting findings were published by Suzuki P. et al. (2010), in a histopathological study using the apical foramen-like communication model with over extrusion of ER, demonstrated the significant ingrown of the connective tissue containing numerous inflammatory cells into the root canal. Additionally, the infiltration of lymphocytes and macrophages with intracytoplasmic ER particles were widely observed [9]. These findings indicate that ER filling material was resorbed and replaced by connective tissue. The latter can explain the findings of this observational study, demonstrating that the level of the material and root resorption in primary molars was different. It indicates the different speed and amount of resorption of the material in comparison to the tissues of the root of the primary molar. The ER tends to be resorbed faster than the root tissues. Additionally, the SEM investigation revealed the non-uniform, lacunary and porous ER surface, visually similar to the resorbed dentin, hypothetically indicating that the material was resorbed by body cells in the same way as the natural hard tissues of the roots.

The different physical and chemical properties of ER were widely tested in numerous studies [14,22]. However, it has been demonstrated, that the solubility of the ER exceeded the maximum weight loss of 3% after immersion in water for 24 h, proposed by ANSI/ADA and ISO standards [15,23]. The increased solubility can have an impact on the speed of resorption of the material when exposed to vital periapical tissues due to the undergoing physiological root resorption. Therefore, if the higher solubility/degradation of ER can have a negative impact on the obturation of root canals in permanent teeth in certain clinical conditions, it can be advantageous in primary teeth, when faster resorption of the filling material is preferable [8].

ER was tested as sealer/filler in clinical trials in permanent teeth with high, acceptable and comparable success rates when used with different obturation techniques [24,25,26]. However, the data about the material as a root canal filler for primary teeth is very limited. Due to acceptable biological properties and demonstrated resorbability, ER can potentially be used as an alternative to commonly used filling materials in primary teeth.

## 5. Conclusions

The SEM and µCT observations of urethane dimethacrylate-based (UDMA) material ER revealed a high potential to be resorbed with no negative influence on the physiological root resorption. The material appears to resorb faster than root tissues and may be suitable as the material for the root canal filling in primary teeth. However, more investigations are needed to support these preliminary observations in conjunction with clinical trials to compare to other available materials.

## Data Availability

Data is contained within the article.
